# The Impact of Intraoperative Fibrinogen Replacement Therapy on the Clinical Outcome of Surgical Therapy for Type A Acute Aortic Dissection

**DOI:** 10.1093/icvts/ivag075

**Published:** 2026-03-04

**Authors:** Makoto Takehara, Takeshi Shimamoto, Kazuhisa Sakamoto, Yu Hidaka, Yuriko Muramatsu, Kenji Minatoya

**Affiliations:** Department of Cardiovascular Surgery, Hamamatsu Rosai Hospital, Hamamatsu, Shizuoka 430-8525, Japan; Department of Cardiovascular Surgery, Tenri Hospital, Nara 632-8552, Japan; Department of Cardiovascular Surgery, Hamamatsu Rosai Hospital, Hamamatsu, Shizuoka 430-8525, Japan; Department of Cardiovascular Surgery, Kyoto University Hospital, Kyoto 606-8507, Japan; Department of Cardiovascular Surgery, Hamamatsu Rosai Hospital, Hamamatsu, Shizuoka 430-8525, Japan; Department of Biomedical Statistics and Bioinformatics, Graduate School of Medicine, Kyoto University, Kyoto 606-8501, Japan; Department of Biomedical Statistics and Bioinformatics, Graduate School of Medicine, Kyoto University, Kyoto 606-8501, Japan; Department of Cardiovascular Surgery, Kyoto University Hospital, Kyoto 606-8507, Japan

**Keywords:** fibrinogen replacement therapy, aortic dissection, coagulopathy

## Abstract

**Objectives:**

Fibrinogen replacement therapy (FRT) using fibrinogen concentrate (FC) may rapidly correct hypofibrinogenaemia; however, its clinical impact remains unclear, and this retrospective study aimed to evaluate the safety and efficacy of FC in surgically treated acute type A aortic dissection (ATAAD) patients.

**Methods:**

A retrospective study analysed 87 consecutive ATAAD patients who underwent emergency surgery. Patients were included in 2 groups: those who received FRT (Group F, *n* = 42) and those who did not (Group C, *n* = 45). Using a mixed model for repeated measures, we calculated the least squares (LS) mean differences between groups for intraoperative and postoperative fibrinogen levels and blood loss using the LS method. We also evaluated short-term surgical outcomes (postoperative complications) and long-term surgical outcomes (overall survival) for each group.

**Results:**

The change in fibrinogen levels during surgery did not differ significantly between groups. Postoperatively, however, the decrease in fibrinogen was smaller in the F group (LSMeans difference: −60.1; 95% confidence interval [CI]: −79.8, −40.4). Intraoperative blood loss was greater in the F group (LSMeans difference: −622 mL; 95% CI, −1037, −208), but no difference was observed in postoperative blood loss (LSMeans difference: −131 mL; 95% CI, −547, 286). Despite a higher proportion of severe cases in the F group, the difference in postoperative complication rates was approximately 10%. The difference in 1-month survival rate was 4.9%, and the difference in 1-year survival rate was 19.5%.

**Conclusions:**

In the surgical treatment of ATAAD, FRT increases fibrinogen levels by approximately 71 mg/dL, achieving adequate haemostasis without increasing adverse outcomes. Additionally, FC is effective in reducing postoperative blood loss in patients with a bleeding tendency whose fibrinogen level is below 150 mg/dL before weaning from cardiopulmonary bypass.

## Graphical Abstract

**Figure:**
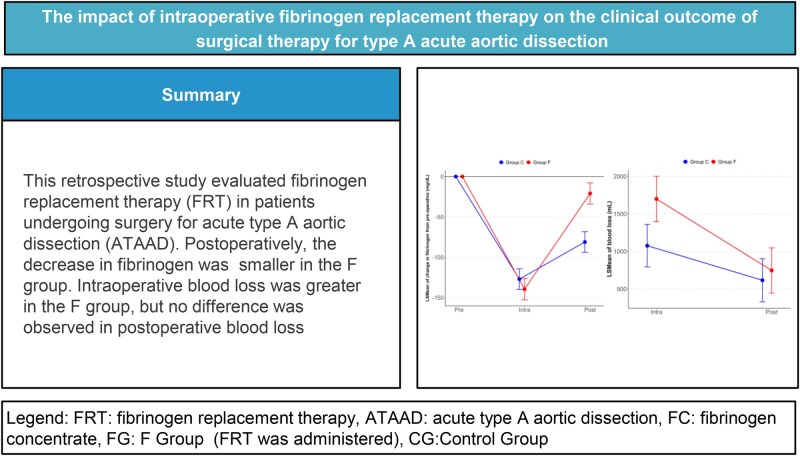

## INTRODUCTION

Perioperative coagulopathy is multifactorial, with contributing factors including hypothermia and other conditions. This phenomenon is associated with increased morbidity and mortality rates among patients undergoing cardiovascular surgery, particularly those diagnosed with acute type A aortic dissection (ATAAD).^1^^,^^2^ The process of perioperative haemostasis is characterized by platelet aggregation, which culminates in the formation of a platelet plug. This initial plug is subsequently stabilized by fibrin, resulting in the development of a robust blood clot. Notably, although factor XIII is the final factor in the coagulation cascade, fibrinogen is the final substrate and exhibits a more rapid decline in concentration compared to other coagulation factors, haematocrit levels, and platelet counts during episodes of intraoperative bleeding.^3^ Consequently, it is now widely acknowledged that the correction of hypofibrinogenaemia is essential in managing major bleeding due to dilutional or consumptive coagulopathy in the context of cardiovascular surgery.^4^ In instances of hypofibrinogenaemia, the mechanism underlying fibrinogen replacement therapy (FRT) utilizing fibrinogen concentrates (FCs) is believed to effectively treat coagulopathy by swiftly restoring normal fibrinogen levels, a process that occurs at a significantly faster rate and with less volume than with fresh frozen plasma (FFP).^5^^,^^6^ However, the ongoing debate regarding the efficacy of FCs in improving clinical outcomes remains unresolved.^7^ Therefore, this retrospective cohort study was designed to investigate the impact of FRT on clinical outcomes in patients with ATAAD, who frequently present with coagulopathy.

## METHODS

### Ethics statement

The study was approved by the Medical Ethics Committee of Hamamatsu Rosai Hospital (Protocol Number: 2021-20; Date of Approval: April 21, 2021) and conducted in accordance with the Declaration of Helsinki and the WMA Declaration of Taipei. As this was a non-invasive observational study in which measures were taken to ensure that subjects could not be identified, the requirement for informed consent was waived.

### Study population

This study included patients who underwent emergency surgery for ATAAD at our hospital between December 2020 and March 2024. The levels of fibrinogen were assessed by the Clauss fibrinogen assay at 3 specific time points: preoperatively, intraoperatively, and postoperatively. FC was administered when the fibrinogen level measured during cardiopulmonary bypass (CPB) approached approximately 150 mg/dL, and the operating surgeon observed a tendency for bleeding in the surgical field, which was characterized by excessive diffuse bleeding from needle holes and soft tissues. Therefore, FC was also administered in cases with a tendency for bleeding, even when the fibrinogen level was above 150 mg/dL. FC was utilized following approval from the Institutional Review Board of Ethics at the hospital. The FC used was Fibrinogen HT I.V. (Japan Blood Products Organization, Japan). The primary protocol during the study period involved the administration of 3 g of FC. At our institution, the criteria for transfusion were as follows: RCC (red blood cell concentrate) transfusion was administered with a target haemoglobin of 10 g/dL; and FFP was administered with a target fibrinogen level of 200 mg/dL. In addition, platelet concentrate (PC) was routinely administered at a dose of 2 units corresponding to a single-donor donation.

### Operative techniques

The CPB priming was performed using 500 mL of hydroxyethyl starch (130 000) and 1000 mL of an extracellular fluid solution, with a priming volume of 1500 mL in all cases. Circulatory arrest was implemented once the bladder or rectal temperature reached 28°C. Upon completion of extracorporeal circulation, heparin was neutralized with protamine, and transfusion therapy was initiated. Protamine (10-15 mg) was administered to neutralize 1000 units of heparin. In Group F, intravenous FC were provided in addition to blood transfusions. In all cases, 1000 mg of tranexamic acid was administered intraoperatively. An intraoperative cell salvage device was utilized during surgery.

### Data collection

All data, including late events, were retrospectively obtained from an integrated electronic medical database system (Fujitsu Limited, Tokyo, Japan) and an automated electronic anaesthesia database (Fortec ORSYS; Philips Electronics Japan, Inc., Tokyo, Japan).

### End-point

The primary end-points of this study are the change in fibrinogen levels from preoperative levels and the amount of postoperative bleeding after FRT. Secondary end-points include short-term surgical outcomes, such as the incidence of postoperative complications, and long-term surgical outcomes such as overall survival.

### Definition

The levels of fibrinogen were assessed preoperatively, intraoperatively, and postoperatively. Postoperative bleeding was defined as the total blood loss measured during surgery as the intraoperative value, and the blood loss drained from the drainage tube within 24 hours as the postoperative value. Cerebral complications were defined as any neurological deficit confirmed by imaging studies during the postoperative hospitalization. Overall survival was defined as the duration from the date of surgery to death from any cause occurring within 1 year postoperatively. Patients without a documented death within 1 year after surgery were censored at 1 year.

### Statistical analysis

Patient background and surgical information were summarized using the median and interquartile range (IQR) or mean and standard deviation (SD) for continuous variables, and frequency and proportion for categorical variables.

For the primary end-point of fibrinogen, outcomes were analysed using a mixed model for repeated measures (MMRM) including group (FC administration, no FC), time point (intraoperative, postoperative), the interaction between group and time, and age, intraoperative RCC dose, and intraoperative FFP dose as confounders, with subject as a random effect. For blood loss, an MMRM including group, time point, the interaction between group and time, and preoperative fibrinogen and platelet values as confounders was used. Least squares (LS) means at each time point and LS mean differences between time points were estimated, together with the corresponding 2-sided 95% confidence intervals (CIs).

For secondary end-points, we calculated the incidence rates of postoperative complications in each group, the differences between groups, and the 95% CIs for these rates. For overall survival, the survival curves for each group were estimated using the Kaplan-Meier method, and we calculated the survival rates at 1 month and 1 year, the differences between groups, and the 95% CIs for them. All statistical analyses were performed with EZR (version 1.61) (Jichi Medical University, Tochigi, Japan), which is a graphical user interface for R (The R Foundation for Statistical Computing, Vienna, Austria), SAS 9.4 (SAS Institute Inc., Cary, NC, USA), and R (version 4.4.2).

## RESULTS

### Patient characteristics and surgical data

**Figure 1.**
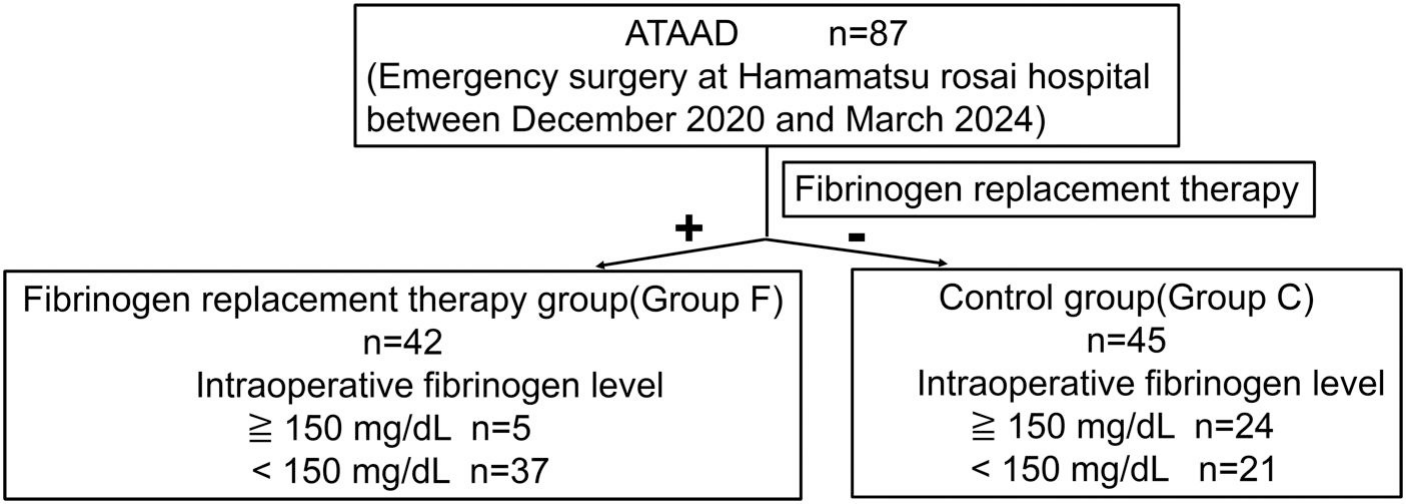
The Flow Diagram of Patients in This Study. Abbreviation: ATAAD, acute type A aortic dissection

The flow diagram for patient inclusion is summarized in **Figure 1**. All 87 cases identified during the study period were included in the analysis. Of these, 42 patients who received FC intraoperatively were included in the FC group (Group F), and 45 patients who did not receive FC were included in the control group (Group C). Group F consisted of 37 patients with fibrinogen levels ≤150 mg/dL and 5 patients with levels >150 mg/dL who were administered FC. Group C consisted of 21 patients with fibrinogen levels ≤150 mg/dL and 24 patients with levels >150 mg/dL who did not receive FC. The characteristics of preoperative patients are summarized in **Table 1**. A total of 87 patients (51 males, 36 females; mean age 71 years) were included. Surgical procedures varied, with 21 ascending, 14 partial arch, and 52 total arch replacements. As shown in **Table 2**, hemi-arch and partial arch replacements were comparable between groups. Most patients in Group F received 3 g of FC intraoperatively. Postoperatively, Group F required more RCC, FFP, and PC than Group C and showed a greater fibrinogen increase, exceeding Group C by more than 71 mg/dL. Other parameters, including the total volume of intravenous fluids administered, the status after weaning from CPB, the doses of heparin and protamine administered intraoperatively, and the activated clotting time (ACT), are summarized in the **Supplementary Table**.

**Table 1.** Preoperative Patients’ Characteristics

	Group F (*N* = 42)	Group C (*N* = 45)
Age, median (IQR), years	71 (60, 79)	71 (59, 78)
Sex, male, *n* (%)	26 (61.9%)	25 (53.1%)
Body weight median (IQR), kg	58.6 (50.1, 69.2)	60.0 (50.0, 74.0)
BMI median (IQR), kg/m²	21.9 (20.9, 26.2)	23.4 (21.7, 27.0)
Past medical history		
HT, *n* (%)	21 (50.0)	28 (62.2)
DM, *n* (%)	2 (4.8)	5 (11.1)
HL, *n* (%)	11 (26.2)	10 (22.2)
CKD or AKI, *n* (%)	21 (50.0)	16 (35.6)
COPD·Asthma, *n* (%)	3 (7.1)	4 (8.9)
OMI, *n* (%)	4 (9.5)	2 (4.4)
Af, *n* (%)	1 (2.4)	2 (4.4)
Circulatory shock, *n* (%)	7 (16.7)	6 (13.3)
CPR, *n* (%)	3 (7.1)	2 (4.4)
Pericardial effusion, *n* (%)	16 (38.1)	12 (26.7)
Previous cerebral infarction, *n* (%)	6 (14.3)	6 (13.3)
Preoperative antiplatelet therapy	4 (9.5)	2 (4.4)
Preoperative anticoagulant therapy	1 (2.3)	2 (4.4)
Preoperative blood test		
Hb median (IQR), g/dL	12.4 (10.7, 13.6)	12.6 (11.5, 13.9)
Platelet count median (IQR),10⁴/μL	16.8 (13.6, 19.8)	19.1 (15.8, 22.6)
PT INR median (IQR)	1.06 (1.00, 1.14)	1.04 (0.98, 1.11)
APPT median (IQR), s	31.4 (28.9, 36.1)	31.5 (27.7, 35.0)
Fibrinogen median (IQR), mg/dL	190 (142, 238)	292 (227, 393)
FDP median (IQR), µg/mL	150 (51.3, 290)	39.4 (8.9, 112)
CRE median (IQR), mg/dL	0.99 (0.86, 1.2)	0.90 (0.76, 1.1)
BUN median (IQR), mg/dL	19 (15, 23.7)	17 (14, 21)
CRP median (IQR), mg/dL	0.075 (0.4, 0.17)	0.25 (0.08, 2.8)
BNP median (IQR), pg/mL	42.7 (24.7, 99.9)	39.2 (20.1, 58.1)

Abbreviations: AKI, acute kidney injury; APPT, activated partial thromboplastin time; BMI, body mass index; BNP, brain natriuretic peptide; BUN, blood urea nitrogen; DM, diabetes mellitus; CKD, chronic kidney disease; COPD, chronic obstructive pulmonary disease; CPR, cardiopulmonary resuscitation; CRE, creatinine; CRP, c-reactive protein; FDP, fibrin/fibrinogen degradation products; HL, hyper lipidaemia; HT, hypertension; IQR, interquartile range; OMI, old myocardial infarction; PT INR, prothrombin time-international normalized ratio.

**Table 2.** Surgical Procedures and Postoperative Data

	Group F (*N* = 42)	Group C (*N* = 45)
Operative procedure		
Hemi arch, *n* (%)	9 (21.4)	12 (26.7)
Partial arch, *n* (%)	5 (11.9)	9 (20.0)
Total arch, *n* (%)	28 (66.7)	24 (53.3)
Intraoperative dose of fibrinogen concentrates		
1 g, *n* (%)	1 (2.4)	–
2 g, *n* (%)	5 (11.9)	–
3 g, *n* (%)	34 (81.0)	–
>3 g, *n* (%)	2 (4.8)	–
Operation progress information		
Operation time, mean (SD), min	303.5 (76.79)	276.6 (56.42)
Extracorporeal time, mean (SD), min	164.7 (45.85)	156.3 (30.73)
Circulatory arrest time, mean (SD),min	41.2 (9.90)	42.0 (7.75)
Haemostasis time, mean (SD),min	49.2 (24.54)	38.7 (24.33)
Postoperative blood transfusion		
RCC, mean (SD), U	4.9 (8.56)	2.3 (2.52)
FFP, mean (SD), U	1.9 (3.61)	1.1 (1.79)
PC, mean (SD), units	0.21 (0.565)	0.09 (0.358)
Fibrinogen increment, mean (SD), mg/dL	118.2 (38.72)	46.9 (38.24)
Postoperative blood test		
Hb median (IQR), g/dL	9.9 (9.1, 10.7)	9.7 (9.1, 9.7)
Platelet count median (IQR),×10³/μL	12.3 (11.2, 14.0)	14.2 (12.8, 17.5)
PT INR median (IQR)	1.2 (1.1, 1.2)	1.1 (1.1, 1.2)
APPT median (IQR), s	38.5 (35.6, 44.6)	37.2 (34.5, 39.5)
Fibrinogen median (IQR), mg/dL	218 (196, 241)	198 (168, 265)

Abbreviations: APPT, activated partial thromboplastin time; FFP, fresh frozen plasma (2 U ≈ 240 mL); IQR, interquartile range; PC, platelet concentrate (1 unit from single donor, ∼2 × 10^{10} platelets; PT INR, prothrombin time-international normalized ratio; RCC, red cell concentrate (2 U ≈ 280 mL); SD, standard deviation; 10 units ≈ 1 international therapeutic dose of ∼2 × 10^{11} platelets).

### Effect of FRT on postoperative fibrinogen levels and blood loss

**Figure 2.**
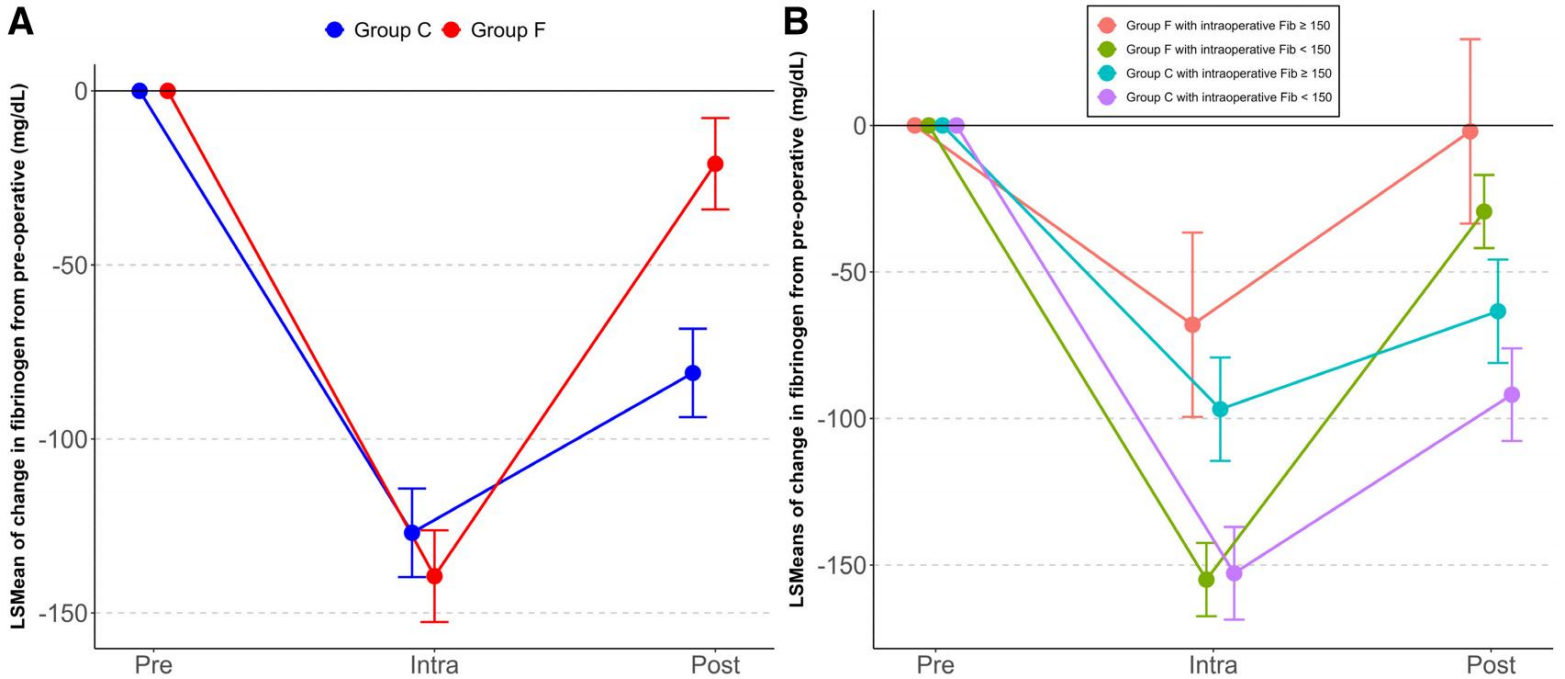
Changes in Fibrinogen Levels from Preoperative Times in the Intraoperative and Postoperative Periods. (A) Changes in the group administered FC and the group not administered FC, (B) changes in subgroups based on intraoperative fibrinogen levels within the group administered FC and the group not administered FC. Abbreviation: LS, least squares

**Figure 2A** shows the changes in fibrinogen levels from preoperative to intraoperative and postoperative in each group. The LSMeans for intraoperative change were −139.4 (95% CI, −152.6, 126.3) for the F group and −127.0 (95% CI, −139.7, −114.3) for the C group. The between-group difference was 12.5 (95% CI, −7.2, 32.1, *P* = .212), indicating no difference. In contrast, the LSMeans for the postoperative change were −21.0 (95% CI, −34.1, −7.8) for Group F and −81.1 (95% CI, −93.8, −68.3) for Group C. The between-group difference was −60.1 (95% CI, −79.8, −40.4), indicating that Group F had a smaller change from preoperative levels. **Figure 2B** shows the results when the group receiving FC administration was divided into 4 subgroups based on intraoperative fibrinogen levels. Among Group F, the subgroup receiving FC despite having an intraoperative fibrinogen level >150 mg/dL had the highest intraoperative and postoperative fibrinogen levels (Intra: −68.1, 95% CI, −99.5, −36.6, Post: −2.0, 95% CI, −33.5, 29.5). The subgroup that was administered FC despite having an intraoperative fibrinogen level of <150 mg/dL had the lowest intraoperative fibrinogen level, but the postoperative level was higher than either of the 2 subgroups in Group C (Intra: −155.0, 95% CI, −167.5, −142.5, Post: −29.4, 95% CI, −41.9, −16.9). Among Group C, both the subgroup with postoperative fibrinogen levels ≥150 mg/dL who were administered FC (Intra: −96.8, 95% CI, −114.5, −79.2, Post: −63.4, 95% CI, −81.1, −45.8) and the subgroup with postoperative fibrinogen levels <150 mg/dL who were not administered FC (Intra: −152.8, 95% CI, −168.6, −137.0, Post: −91.9, 95% CI, −107.7, −76.1) had lower postoperative fibrinogen levels than either subgroup of Group F.

**Figure 3.**
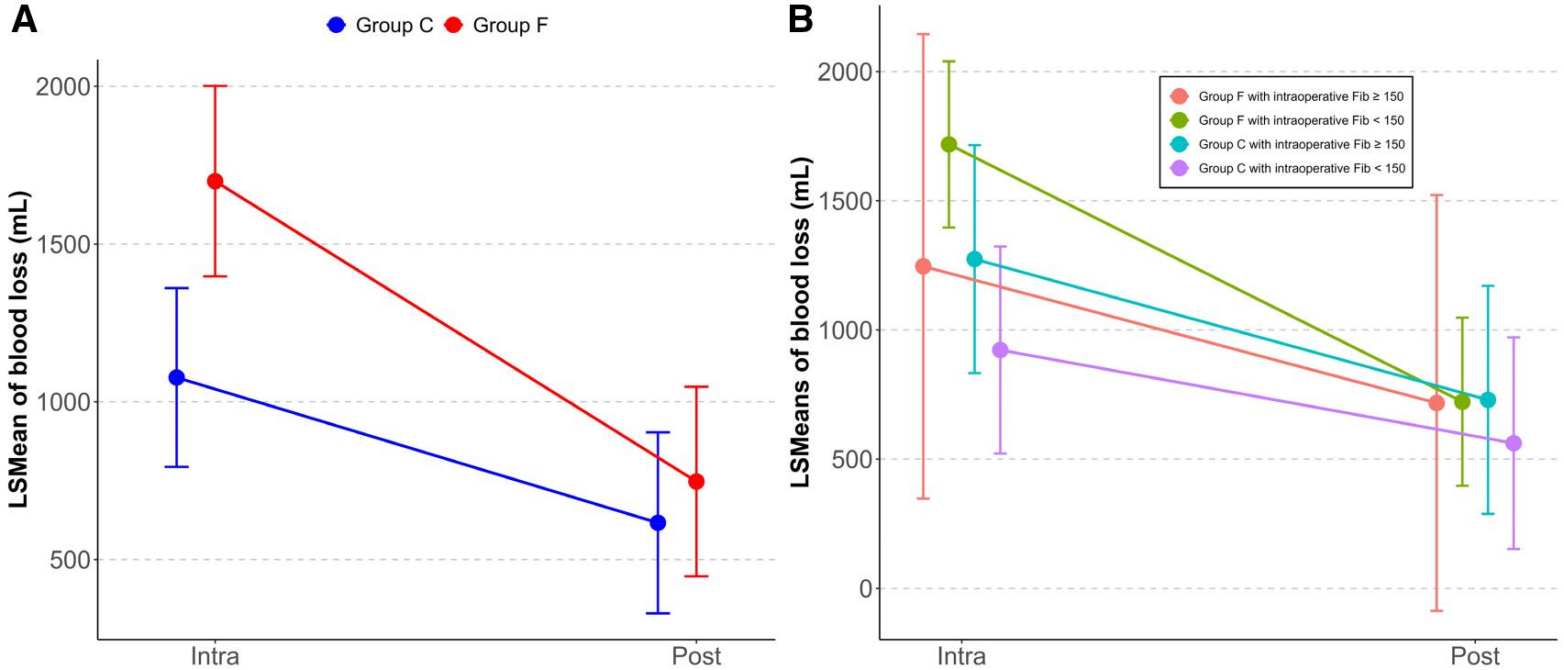
Blood Loss Intraoperative and Postoperative Period. (A) Changes in the group administered FC and the group not administered FC, (B) changes in subgroups based on intraoperative fibrinogen levels within the group administered FC and the group not administered FC. Abbreviation: LS, least squares

**Figure 3A** shows changes in blood loss from the intraoperative to postoperative periods in each group. The LSMeans for intraoperative blood loss were 1077 mL (95% CI, 794, 1360) for the F group and 1699 mL (95% CI, 1397, 2001) for the C group, with a between-group difference of −622 mL (95% CI, −1037, −208), indicating greater intraoperative blood loss in the F group. Postoperatively, LSMeans were 616 mL (95% CI, 329, 903) for the F group and 747 mL (95% CI, 447, 1048) for the C group, with a between-group difference of −131 mL (95% CI, −547, 286), showing no difference.

**Figure 3B** shows subgroup analyses of blood loss. The F group with intraoperative fibrinogen <150 mg/dL had the highest intraoperative blood loss (1718 mL; 95% CI, 1396, 2039), whereas values for Group F with intraoperative fibrinogen ≥150 mg/dL (1246 mL; 95% CI, 347, 2145), Group C with intraoperative fibrinogen ≥150 mg/dL (1274 mL; 95% CI, 833, 1715), and Group C with intraoperative fibrinogen <150 mg/dL (922 mL; 95% CI, 522, 1323) were lower. Postoperatively, blood loss was similar across subgroups: F group with intraoperative fibrinogen <150 mg/dL (722 mL; 95% CI, 397, 1048), F group with intraoperative fibrinogen ≥150 mg/dL (717 mL; 95% CI, −87, 1522), C group with intraoperative fibrinogen ≥150 mg/dL (729 mL; 95% CI, 288, 1170), and C group with intraoperative fibrinogen <150 mg/dL (562 mL; 95% CI, 152, 971). To address potential surgeon-related decision-making bias, sensitivity analyses using MMRMs incorporating surgeon as an additional random effect were performed, confirming the robustness of the main findings for fibrinogen levels and blood loss.

In summary, Group F showed smaller changes in fibrinogen levels from preoperative to postoperative periods. In the subgroup analysis, the subgroups in Group C showed lower postoperative fibrinogen levels than the subgroups in Group F, regardless of intraoperative fibrinogen levels. Intraoperative blood loss was greater in Group F, but the difference disappeared postoperatively. In the subgroup analysis, the subgroup in Group F with intraoperative fibrinogen levels <150 mg/dL had the greatest intraoperative blood loss, whereas postoperative blood loss did not differ among subgroups.

### Short- and long-term surgical outcomes

Short- and long-term surgical outcomes are shown in **Table 3**. For short-term surgical outcomes (postoperative complications), the number of patients requiring re-exploration for bleeding was 3 cases (7.1%) in Group F and 1 cases (2.2%) in Group C, with a group difference of 4.9% (95% CI, −5.5, 17.5). Patients who developed postoperative cerebral stroke were 6 cases (14.3%) in Group F and 3 cases (6.7%) in Group C, with an inter-group difference of 7.6% (95% CI, −6.1, 23.2). Additionally, 1 patient (2.4%) in Group F and no patients in Group C developed pulmonary thromboembolism, with a between-group difference of 2.4% (95% CI, −5.8, 12.6). Other thromboembolic events occurred in 8 patients (19.1%) in Group F and 3 patients (6.7%) in Group C, with a between-group difference of 12.4% (95% CI, −2.1, 28.1).

**Table 3.** Secondary End-points: Surgical Outcomes

	Group F (*N* = 42)	Group C (*N* = 45)	Group differences (95% CI)
Short-term surgical outcomes (postoperative complications)			
Re-exploration for bleeding, *n* (%)	3 (7.1)	1 (2.2)	4.9% (−5.49%, 17.49%)
Cerebral complications, *n* (%)	6 (14.3)	3 (6.7)	7.6% (−6.09%, 23.21%)
Pulmonary thromboembolism, *n* (%)	1 (2.4)	0 (0.0)	2.4% (−5.77%, 12.58%)
Other thromboembolic or ischaemic event, *n* (%)	8 (19.1)	3 (6.7)	12.4% (−2.14%, 28.08%)
Long-term surgical outcomes (overall survival)			
One-month survival rate (95% CI), %	92.9 (79.5, 97.6)	97.8 (85.3, 99.7)	−4.9 (−13.8, 4.0)
One-year survival rate (95% CI), %	73.8 (57.7, 84.6)	93.3 (80.7, 97.8)	−19.5 (−34.7, −4.4)

Abbreviation: 95% CI, 95% confidence interval.

**Figure 4.**
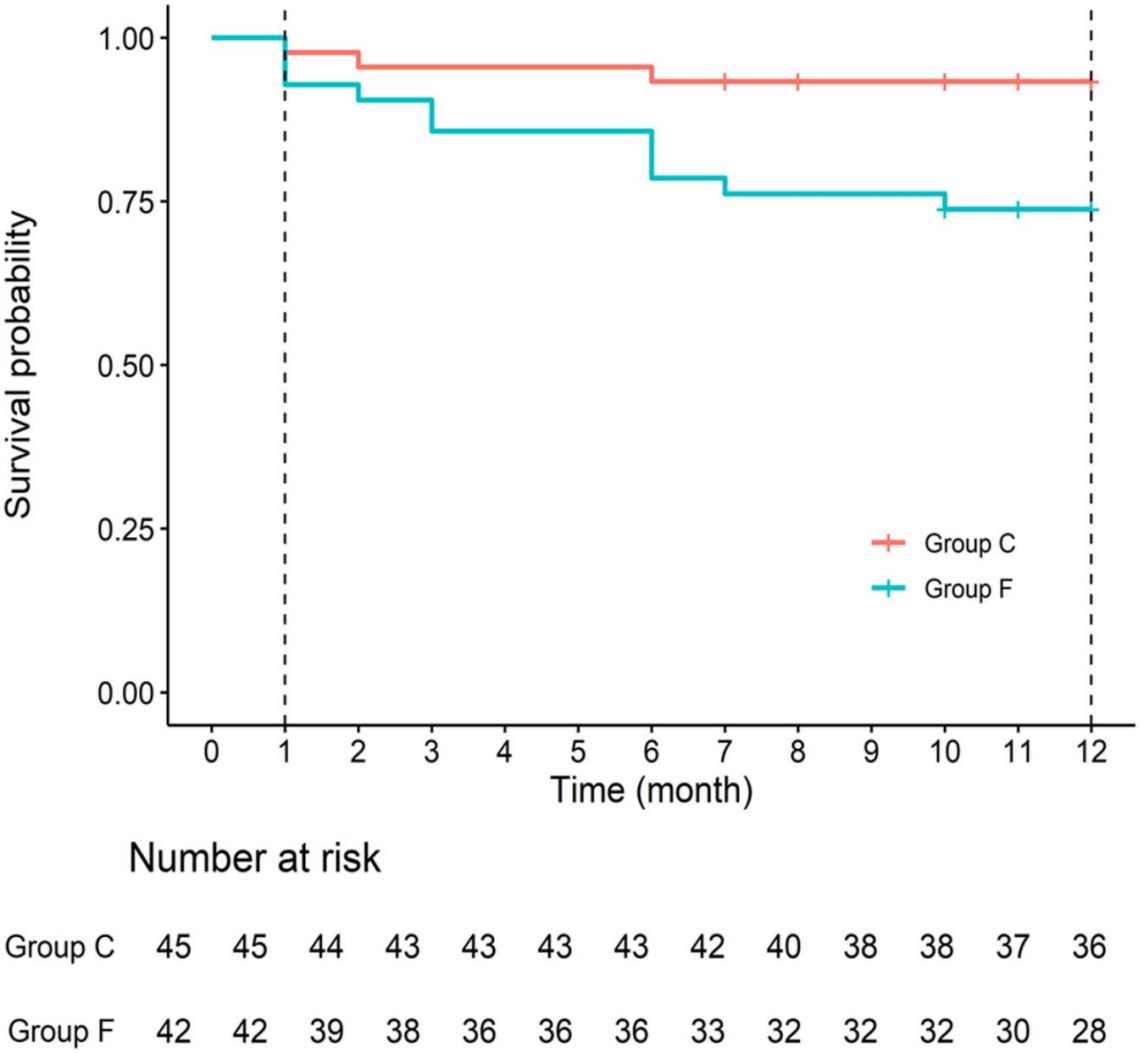
Kaplan-Meier Curves for Overall Survival by FC Administration Group

**Figure 4** shows the Kaplan-Meier curves for long-term surgical outcomes (overall survival) in the 2 groups. The 1-month survival rate was 92.9% (95% CI, 79.5-97.6) in Group F and 97.8% (95% CI, 85.3-99.7) in Group C, with a group difference of −4.9% (95% CI, −13.8 - −4.0). The 1-year survival rate was 73.8% (95% CI, 57.7-84.6) for Group F and 93.3% (95% CI, 80.7-97.8) for Group C, with a difference between groups of −19.5% (95% CI, −34.7 - −4.4).

## DISCUSSION

The results of this study suggest that FRT during surgery is effective in correcting postoperative fibrinogen levels and reducing postoperative bleeding in patients of ATAAD.

The postoperative fibrinogen level in the group that received FRT was higher than in the group that did not receive it, and the amount of postoperative bleeding was nearly equal between the 2 groups. In this study, the decision to perform FRT was made by the surgeon based on intraoperative laboratory values. Although the group that received FRT had lower preoperative and intraoperative fibrinogen levels, the difference in postoperative fibrinogen levels disappeared, suggesting a corrective effect of FRT on fibrinogen levels. Similar results were obtained in analyses that accounted for potential confounding effects of the surgeon’s treatment decisions.

Subgroup analysis showed that the group not receiving FRT had a greater decrease in postoperative fibrinogen levels regardless of intraoperative fibrinogen levels, suggesting a compensatory effect of FRT.

It is plausible to suggest that FRT plays a critical role in mitigating coagulopathy at the onset of the withdrawal from CPB, thereby preventing consumption coagulopathy associated with subsequent bleeding events, as has also been demonstrated in other reports involving thoracic aortic surgery patients.^8^ Hypofibrinogenaemia is known to induce coagulopathy during the surgical management of ATAAD, particularly in the context of CPB, hypothermia, and circulatory arrest.^9^ In cases of severe coagulopathy, one would typically anticipate an increase in operative duration, haemostasis time, and reoperation rates due to bleeding. However, the current study did not reveal any differences in these parameters between the groups with and without FRT. This finding may be attributed to the efficacy of FRT in ameliorating coagulopathy at the initial phase of CPB withdrawal. Furthermore, the observed increase in intraoperative bleeding within the FRT group, assessed in conjunction with bleeding prior to the administration of FRT, suggests that this group may have experienced more pronounced coagulopathy rather than indicating an inadequate effect of FRT. It has been reported that patients with lower fibrinogen levels after cardiac surgery are at a higher risk of bleeding and re-exploration,^10^ indicating that hypofibrinogenaemia reflects a more severe coagulopathic state.

Although the F group showed greater intraoperative blood loss, there was no difference in the 24-hour postoperative drain output after FC administration. This finding suggests a direct haemostatic effect of FC.

Furthermore, in this study, no differences were observed between the FRT group and the non-FRT group in major end-points such as thrombosis or mortality.

Thus, FRT appears to be a safe intervention that effectively corrects fibrinogen deficiency and yields favourable clinical outcomes in patients with severe coagulopathy and hypofibrinogenaemia. The optimal dosage of fibrinogen replacement and the ideal target fibrinogen level for the reduction of blood loss remain to be determined.^11^ In patients undergoing cardiovascular surgery, recent studies have predominantly utilized a fibrinogen replacement dose ranging from 2 to 4 g^12–17^; however, some investigations have employed higher doses of 6 to 8 g.^11^^,^^18^ In the current study, administration of 3 g of fibrinogen resulted in an approximate increase of 71 mg/dL in fibrinogen levels among patients with hypofibrinogenaemia. Notably, the postoperative fibrinogen levels in these patients did not differ from those in patients without hypofibrinogenaemia who did not receive FRT. Fibrinogen supplementation was provided to patients exhibiting bleeding tendencies, irrespective of their baseline fibrinogen levels, and was also administered in certain cases where fibrinogen levels were not critically low. This raises the possibility that fibrinogen supplementation may not be effective in patients without hypofibrinogenaemia. In the subgroup analysis, patients in the FRT group with fibrinogen levels <150 mg/dL had greater intraoperative blood loss; however, the difference in postoperative bleeding was no longer observed, suggesting that an intraoperative fibrinogen level of 150 mg/dL may represent an appropriate threshold. In cardiac surgery, intraoperative hypofibrinogenaemia (fibrinogen level <150 mg/dL) has been identified as an independent risk factor for excessive postoperative bleeding, as evidenced by a 24-hour chest drainage volume exceeding 500 mL.^19^ Given that the findings of the present study, which employed a basic dose of 3 grams, demonstrated adequate control of bleeding tendencies, any potential increase in dosage should be carefully considered in relation to safety. Taking into account the possible adverse effects associated with excessive FRT, such as the risk of thromboembolism,^20^ the use of 3 grams appears to be a judicious choice based on the current study’s results, which successfully avoided an increase in transfusion volume without a corresponding rise in embolic events.

FC offers several advantages over FFP transfusion, including a lower volume load, reduced risk of transfusion-related adverse events and viral transmission, and faster preparation.^21^ These benefits suggest that FC may be a viable therapeutic option in the surgical management of ATAAD, where prompt intervention is critical.

### Limitations

This study had several limitations. First, the single-centre and retrospective design with a relatively small sample size increases the risk of bias and limits the generalizability of our findings. Therefore, multivariate analyses and mixed models for repeated measures were used to evaluate the association between FC administration and clinical outcomes: however, the factors adjusted for as confounders in this study may not be sufficiently comprehensive, and unmeasured confounders may exist. Consequently, future studies employing more confirmatory designs are necessary. Second, the decision to administer FC and other blood products (red blood cells, FFP, and platelets) was ultimately left to the discretion of the attending surgical and anaesthesia teams, which may have introduced surgeon- or provider-dependent bias. Although sensitivity analyses incorporating surgeon as a random effect yielded results consistent with the main analyses, these approaches cannot completely eliminate confounding by indication.

Additionally, another possible limitation of this study was the use of multiple criteria for FFP transfusions. Further confirmatory studies using predefined, clearly specified transfusion and FC administration criteria are needed to validate our observations.

## CONCLUSION

In the surgical treatment of ATAAD, FRT increases fibrinogen levels by approximately 71 mg/dL, achieving adequate haemostasis without increasing adverse outcomes. FC is effective in reducing postoperative blood loss in patients with a bleeding tendency whose fibrinogen level is below 150 mg/dL before weaning from CPB.

Importantly, this intervention does not appear to affect the rates of re-exploration for bleeding or mortality among patients undergoing surgical treatment for ATAAD.

## Supplementary Material

ivag075_Supplementary_Data

## Data Availability

All relevant data are within the manuscript and its **Supporting Information Files**.

## ABBREVIATIONS

ACT: activated clotting time
Af: atrial fibrillation
AKI: acute kidney injury
APPT: activated partial thromboplastin time
ATAAD: acute type A aortic dissection
BMI: body mass index
BNP: brain natriuretic peptide
BUN: blood urea nitrogen
CKD: chronic kidney disease
CPB: cardiopulmonary bypass
CPR: cardiopulmonary resuscitation
CRE: creatinine
CRP: c-reactive protein
DM: diabetes mellitus
FC: fibrinogen concentrate
FDP: fibrin/fibrinogen degradation products
FET: frozen elephant trunk
FFP: fresh frozen plasma
FRT: fibrinogen replacement therapy
HL: hyper lipidemia
HT: hyper tension
IQR: interquartile range
LS: least squares
MMRM: mixed model for repeated measures
OMI: old myocardial infarction
PC: platelet concentrate
PT INR: prothrombin time-international normalized ratio
RCC: red blood cell concentrate
SD: standard deviation
