# Supplementary material for: The Impact of Intraoperative Fibrinogen Replacement Therapy on the Clinical Outcome of Surgical Therapy for Type A Acute Aortic Dissection
Source: Interdiscip Cardiovasc Thorac Surg. 2026 Mar 4;41(3):ivag075. doi: 10.1093/icvts/ivag075 (PMC13006199; doi:10.1093/icvts/ivag075)
Supplement: ivag075_Supplementary_Data [file ivag075_supplementary_data.docx]

Supplementary table: Details of CPB management and post-weaning status

|  | Group F (N=42) | Group C (N=45) |
| --- | --- | --- |
| Intraoperative | | |
| fluids (mL) | 4834(2836, 6557) | 3033 (1945, 5187) |
| Heparin (U) | 22000 (20000, 25000) | 25000 (21000, 30000) |
| Protamine (mg) | 180(140, 200) | 185 (130, 200) |
| ACT(pre CPB) (S) | 530 (470,808) | 515 (475,586) |
| ACT(CPB) (S) | 954 (759,1500) | 842 (706,1043) |
| Bladder/ Rectal temperature (℃) | 35.5(35.2,36) | 35.5 (35.3,36) |
| Post-CPB | | |
| ACT (S) | 137 (129,147) | 127 (120,134.5) |
| Bladder/ Rectal temperature (℃) | 26.4(25.9,27.1) | 26.3 (25.5,26.9) |
| pH | 7.326 (7.278,7.366) | 7.316 (7.2685,7.3365) |
| Na (mEq) | 134 (132,135) | 132 (130,134) |
| Cl (mEq) | 108 (107,109) | 107 (105,109) |
| K (mEq) | 4.78 (4.33,5.09) | 4.93(4.52,5.47) |
| Ca (mmol/L) | 1.04 (0.96,1.10) | 1.11(1.04,1.13) |
